# Comments on the article “Olanzapine as a prophylactic antiemetic for preventing postoperative nausea and vomiting after general anesthesia: A systematic review and meta-analysis”

**DOI:** 10.1016/j.clinsp.2024.100569

**Published:** 2025-01-03

**Authors:** Pedro Fernandes Ribeiro, Bianca Ramos, Tallys A. Suzuki, Giovanna Uyeda

**Affiliations:** Faculdade de Medicina da Universidade de São Paulo (FMUSP), São Paulo, SP, Brazil

The authors have read the article “Olanzapine as a prophylactic antiemetic for preventing postoperative nausea and vomiting after general anesthesia: A systematic review and meta-analysis”, published by Grigio et al.^1^ in the Clinics journal with great interest. Would also like to discuss some issues within the decisions made in this article.

This systematic review and meta-analysis aimed to evaluate the efficacy and safety of prophylactic olanzapine as an antiemetic in adult patients who underwent general anesthesia. The authors started their study with 261 manuscripts, and, with solid criteria, three studies were selected for final analysis ‒ all of them RCTs.

The authors would like to commend the authors’ use of AI tools, such as^2^ Rayyan, for initial screening of studies capable of inclusion within the review, as well as their use of^3^ tools suggested by the National Heart, Lung and Blood Institute (NHLBI) to scrutinize each of the studies for risk of bias assessment. On top of it, the authors were clear about the remaining flaws of their analysis, such as the high risk of bias in one of the studies^4^ and heterogeneity of the data not always coming from the primary outcomes of the studies. Overall, they seem to have followed general and classic recommendations for identifying and avoiding biases as summarized by Pannucci et al.^5^

In light of what the authors discussed above, it is clear to us that the authors are well aware of how data should be handled in such analysis. Nevertheless, the authors were left curious about why these particular studies were selected and would like to have a better understanding of a decision made by the authors when including them in their meta-analysis. Despite their acknowledgment of heterogeneity in how each of the analyzed studies^4^^,^^6^^,^^7^ defined their respective interventions, comparisons, time of measure, and the measured outcome, why did the authors choose to include them all in the same analysis? Wouldn't this decision overestimate the effects of the prophylactic use of olanzapine as an antiemetic?

Also, supposing there were more studies available, worthy of selection for analysis after bias assessment, with homogeneous defined interventions, comparisons, measured outcomes, and so on, would the authors still maintain the three studies in the meta-analysis (i.e., would the authors handle the analysis differently, for example, excluding one or some of such studies)?

Finally, the authors would like to commend that papers such as this one provide substantial ground for further research. It is, after all, the first systematic review and meta-analysis regarding the theme, a very well-made one, and, despite limitations discussed above, will certainly be an important starting point for future studies.

## Declaration of competing interest

The authors declare no conflicts of interest.
